# Simultaneous adsorption of As(III) and Cd(II) by ferrihydrite-modified biochar in aqueous solution and their mutual effects

**DOI:** 10.1038/s41598-022-09648-1

**Published:** 2022-04-08

**Authors:** Xiaosong Tian, Qing Xie, Guanqun Chai, Guanghui Li

**Affiliations:** 1 College of Resources, Environment and Safety, Chongqing Vocational Institute of Engineering, Chongqing, 402260 China; 2 Chongqing Engineering Research Center for Soil Contamination Control and Remediation, Chongqing, 400067 China; 3 Interdisciplinary Research Center for Agriculture Green Development in Yangtze River Basin, College of Resources and Environment, Southwest University, Chongqing, 400715 China; 4 Institute of Soil and Fertilizer, Guizhou Academy of Agricultural Sciences, Guiyang, 550006 China

**Keywords:** Environmental chemistry, Environmental impact

## Abstract

A simply synthetic ferrihydrite-modified biochar (Fh@BC) was applied to simultaneously remove As(III) and Cd(II) from the aqueous solution, and then to explore the mutual effects between As(III) and Cd(II) and the corresponding mechanisms. The Langmuir maximum adsorption capacities of As(III) and Cd(II) in the single adsorbate solution were 18.38 and 18.18 mg g^−1^, respectively. It demonstrated that Fh@BC was a potential absorbent material for simultaneous removal of As(III) and Cd(II) in aqueous solution. According to the XRF, SEM–EDS, FTIR, XRD, and XPS analysis, the mechanisms of simultaneous removal of As(III) and Cd(II) by Fh@BC could be attributable to the cation exchange, complexation with R-OH and Fe-OH, and oxidation. Moreover, the mutual effect experiment indicated that Cd(II) and As(III) adsorption on Fh@BC in the binary solution exhibited competition, facilitation and synergy, depending on their ratios and added sequences. The mechanisms of facilitation and synergy between Cd(II) and As(III) might include the electrostatic interaction and the formation of both type A or type B ternary surface complexes on the Fh@BC.

## Introduction

Cadmium (Cd) and arsenic (As) are two of the most common heavy metal(loid)s found in the water and soil environment. Cd and As pollution pose tremendous risks to human health due to their high toxicity^1^, and can result in enormous economic losses^2^. Therefore, the remediation of Cd and As co-contaminated environments have become an urgent task^3–5^. Cd often coexists with As in the water/soil environment^6^, but each exhibits different geochemical behaviours^7,8^. Therefore, the conventional measures that involve the regulation of pH or Eh are difficult to remediate the As and Cd co-contaminated environments^9^. The removal and immobilization of As and Cd in the water/soil environment are the main approaches for environmental remediation, with adsorption being one of the most critical mechanisms involved in this process^3,5,10^. Generally, environmental remediation materials include clay minerals, metal oxides, phosphate compounds, lime materials, compost, and biochar^11^.

Biochar, a carbon-rich product derived from biomass pyrolysis under an oxygen-limited atmosphere, has attracted an increasing attention in recent years^12–14^. The massive production of agricultural wastes^10,15^, restaurant garbages^16^, sewage sludges^17,18^, manures^19,20^, and wood chips^21,22^ provide an abundant source for biochar preparation. Moreover, the unique inherent properties of biochar (such as large surface area, high porosity, active surface functional groups and favorable cation-exchange capacity) make them have possible to remediate the contaminated water and soil environments^23,24^. Many studies have verified that biochar or modified biochar is effective for the removal of heavy metal(loid)s (including Hg^25^, Pb^26^, Cr^27^, Cd^26,28^, and As^29,30^), as well as eutrophication elements^31,32^ and organic pollutants^33,34^. Because of the negative charge of pristine biochar^14^, and different geochemical behaviors of As and Cd (*e.g.*, zeta potentials, pH-dependence, and Eh-dependence)^8^, the biochar application to the As and Cd co-contaminated water/soil environment has been limited.

Recently, several studies have begun to explore the efficiencies and mechanisms of simultaneous adsorption of As and Cd by modified biochars in aqueous solution^9,33^. To enhance the adsorption capacity of biochar for As and Cd, the incorporation of metal (hydr)oxides has received a great deal of attention^33,35,36^, especially the incorporation of iron (hydr)oxides such as goethite^33^, hematite^29^, magnetite^3,9,15,37^, zero-valent iron (ZVI)^4,38^ and iron-bearing mixture^35,39^. Among the iron (hydr)oxides, ferrihydrite is one of the most critical natural mineral adsorbents that could be used to control both cation and oxyanion contaminants in the soil and water environment; However, ferrihydrite has not received significant attention. Ferrihydrite, with a high specific surface (100–700 m^2^ g^−1^) and pH_zpc_(7.8–8.8), is a critical component of the soil sorption complex^40^. Several studies have demonstrated that, among the iron (hydr)oxides, ferrihydrite has the highest adsorption capacity for arsenic (2.1 mmol g^−1^)^41^. Ferrihydrite can form a bidentate or monodentate complex with As-containing compounds and a monodentate complex with Cd^2+^^42^, and can act as a natural Fenton reagent that can oxidize As(III) to As(V)^43^. Huang et al.^44^ further studied the oxidation for As(III) and adsorption for As(III) and As(V) in water environment using the ferrihydrite-loaded biochar as a Fenton-like reagent, but cations such as Cd^2+^ were not taken into account together. From the above, we speculate that ferrihydrite-loaded biochar might adsorb or remove As and Cd in aqueous, and then the ferrihydrite-loaded biochar was assembled to perform the removal capacities of As and Cd and explain the corresponding mechanisms.

Recently, several studies have evaluated As and Cd adsorption on modified biochar in aqueous solution^15,21,22,45^, a few studies have begun to investigate the mutual effect and mechanism between As and Cd in the dual adsorbates system^4,9,10^. However, there are some contradictory results. For example, Cd(II) and As(V) had a competitive effect in the binary solution with the concentration of > 100 mg L^−1^^10^; on the contrary, As may also promote Cd adsorption in the binary solution in previous studies^4,5^. Furthermore, Wu et al.^9^ demonstrated that the presence of As(III) facilitated Cd(II) adsorption about 3–16% while Cd(II) suppressed As(III) adsorption about 15–33%. It can be seen that the interaction between Cd and As in the dual adsorbents system is still controversial. Therefore, it is necessary to further explore the interaction of Cd and As, such as the condition or mechanism of occurrence.

In this study, we proposed a facile, inexpensive and effective ferrihydrite-modified biochar (Fh@BC) for the simultaneous removal of As and Cd in aqueous solution. The objectives of this study were to (1) verify the efficiency of Fh@BC for the simultaneous removal of Cd(II) and As(III); (2) evaluate the adsorption behaviors of Fh@BC with batch adsorption experiments; and (3) elucidate the mutual effect and removal mechanism of As(III) and Cd(II) with the characterizations of Fh@BC.

## Materials and methods

### Materials

Rape straw was collected from Ziyang City (Sichuan Province, China) and was used as feedstock for the production of pristine biochar (PBC). Fe(NO_3_)_3_·9H_2_O and KOH (AR grade) were selected to modify the PBC; NaAsO_2_ and Cd(NO_3_)_2_·4H_2_O) (AR grade) were used to prepare the stock solutions for the batch experiment. All reagents were from Reagent Co. Ltd. All solutions were prepared with ultrapure water (18.2 MΩ).

### Preparation of ferrihydrite-modified biochar

Rape straw was air-dried and ground to < 5 mm. The feedstock was pyrolyzed in a furnace in the N_2_ atmosphere. The pyrolysis temperature was increased to 400 °C at a rate of 5 °C min^−1^ and then maintained at 400 °C for 2 h. After cooling to room temperature, the biochar was washed with the deionized water and filtered using a 300-mesh sieve. The biochar that passed through the sieve was collected and was referred to as PBC.

The ferrihydrite-modified biochar (Fh@BC) was synthesized according to a previously reported method^44^ with some modifications. First, the dried PBC of one gram was submerged in the 0.1 M Fe(NO_3_)_3_solution of 50 mL. The suspension with pH < 2 was vibrated in a thermostatic shaker (25 °C) at 180 rpm for 24 h. Then,1 M KOH solution was added into the suspension to adjust the pH to 7.0 ± 0.1, which is the same as that used in the synthesis of pure ferrihydrite. The suspension was stirred vigorously using a magnetic stirrer at 600 rpm for 30 min at room temperature (~ 25 °C). The modified biochar was continuously washed by the deionized water until the conductivity of the aqueous solution was less than 50 μS cm^−1^, and then was separated using a 300-mesh sieve. Fh@BC and Fh were freeze-dried and stored at 4 °C in the dark for later experiments^42^.

### Batch experiments

Batch experiments were performed to evaluate the adsorption capacity and performance of the adsorbent for As(III) and Cd(II). The background electrolyte of the reaction system was 0.01 M NaNO_3_. Briefly, all batch adsorption experiments were performed with 50 ± 0.1 mg PBC or Fh@BC in a 20 mL solution. The suspension was placed into 50 mL centrifuge tubes and then vibrated in a thermostatic shaker at a velocity of 180 rpm at 25 °C. Afterward, the suspension was filtered through 0.45 µm disposable filters for subsequent determination.

The adsorption kinetics experiments included single and binary solutions. In the single adsorbate solution, the adsorption kinetics experiment was conducted in 10 mg L^−1^ As(III) or Cd(II) solution at pH= 7.0 ± 0.1 for 24 h of oscillation (denoted as “As” or “Cd”). In the binary solution, two approaches were adopted, as follows: (1) 20 mg L^−1^ As(III) solution and 20 mg L^−1^ Cd(II) solution were mixed in equal volume and the solution pH was regulated to 7.0 ± 0.1, and the theoretical concentrations of As(III) and Cd(II) in the mixed solution was 10 mg L^−1^ (denoted as “Cd and As”); and (2) As(III) or Cd(II) solution was added into the Cd(II) or As(III) solution successively after a 24 h pre-equilibrium reaction, and the concentration of Cd(II) and As(III) in the binary solution was about 10 mg L^−1^ (denoted as “Cd + As” for Cd(II) or “As + Cd” for As(III)). The sequential As(III) or Cd(II) in the binary solution was determined after a 24 h equilibrium and denoted as “Cd + As” for As(III) or “As + Cd” for Cd(II). At the appropriate time, the suspension was filtered for subsequent determination. In this study, pseudo-first-order (PFO) kinetic model and pseudo-second-order (PSO) kinetic model were adopted to describe the adsorption rates^33,46^, as shown in the Supplementary Information (SI. 1).

Adsorption isotherm experiments also evaluated single and binary solutions. For the single solution, the isotherm experiment was conducted with various concentrations of As(III) or Cd (II) solution (pH = 7.0 ± 0.1), ranging from 1 to 200 mg L^−1^. For the binary solution, two approaches were considered: (1) various concentrations of As(III) or Cd(II) solution (pH = 7.0 ± 0.1), ranging from 1 to 200 mg L^−1^, were mixed simultaneously (denoted as “Cd and As”); and (2) 5 mg L^−1^ As (III) or Cd(II) solution was successively added into the pre-equilibrium solution with the Cd(II) or As(III) concentration ranging from 1 to 200 mg L^−1^ (denoted as “Cd + As” or “As + Cd”). The Langmuir and Freundlich models were adopted for data fitting for the adsorption isotherm experiments of Cd(II) and As (III)^33,47^, as shown in SI. 2.

To further understand the adsorption performance and mechanisms of Fh@BC for Cd(II) and As(III), solution pH, competition ions, and oxidation capacity of Fh@BC for As(III) were also examined. The detailed methods are described in SI. 3, 4.

### Measurements and characterization

Element contents of PBC and Fh@BC before and after adsorption were determined by X-ray fluorescence spectroscopy (XRF; Cadence, XOS, USA). Morphology and element features of PBC and Fh@BC were characterized by a scanning electron microscope with energy dispersion spectrometry (SEM–EDS; SU8020, Hitachi, Japan). N_2_ adsorption–desorption isotherms were determined using an ASAP 2460 analyzer (Micromeritics Instrument, USA). The specific surface areas of PBC and Fh@BC were calculated by the Brunauer–Emmett–Teller (BET) method. Surface functional groups of PBC and Fh@BC were evaluated according to the Fourier transform infrared (FTIR) spectra obtained with a Nicolet IS10 instrument (Thermo Fisher Scientific, USA) with a scanning range of 4000–400 cm^−1^. X-ray diffraction (XRD) was used to identify the crystalline structures of PBC and Fh@BC at a scanning rate of 6° min^−1^ and a 2θ range of 10°–90°, using a BRUCKER D8 (Bruker, Germany). The chemical states of the elements were evaluated by X-ray photoelectron spectroscopy analysis (XPS) using a Thermo ESCALAB 250Xi (Thermo Fisher Scientific, USA). All spectra were calibrated with the binding energy of the C1s peak at 284.8 eV. The concentrations of total As or As(III) in solution were quantified using atomic fluorescence spectrometry (AFS; PERSEE, China). The concentrations of Cd(II) and iron (Fe) in the aqueous solution were quantified by flame atomic absorption spectrometry (AAS; PERSEE, China). Solution pH was measured using a pH meter (H160NP, Hach, USA).

## Results and discussion

### Characterization of Fh@BC

As illustrated in Fig. S1, the pore size of Fh@BC was about 10 μm. From Fig. S1d, it can be seen that iron (hydr)oxides with ~ 30 nm spherical particles covered the surface or pores of Fh@BC. According to Table S1, iron (hydr)oxides loaded on the Fh@BC. Moreover, the BET of Fh@BC increased from 3.76 to 4.13 m^2^ g^−1^ during the synthesis process in the Fe (NO_3_)_3_ solution (pH < 2).

The FTIR analysis was shown in Fig. S2a. The broadband of Fh@BC near ~ 3340 cm^−1^ was strengthened and was attributed to the stretching vibration of FeO-H^33,48^ or RO-H^32,49,50^, This indicated that –OH was induced on the PBC during the modified procedure. The peak at 1591 cm^−1^ (assigned to aromatic C=C or C=O groups of the carboxyl) weakened after interaction with Fe^3+^, implying that aromatic C=C or C = O groups of the carboxyl unit were consumed during the modification process^26^. Fh@BC showed a new characteristic peak at 1376 cm^−1^ resulting from the stretching Fe-OH vibrations^48^. As mentioned above, iron (hydr)oxides formed on the surface or in the pores of PBC.

The XRD pattern of Fh@BC is illustrated in Fig. S2b. A strong and broad peak at approximately 23° indicated that the crystal plane resulted from typical disordered glassy polymers of carbon^51^. The CaCO_3_ diffraction peak of Fh@BC became weak and even disappeared after the modification, with a decreased peak at 873 cm^−1^ on FTIR; this result is consistent with the reduced calcium contents of Fh@BC in Table S1. In contrast, the related characteristic peaks of iron (hydr)oxides in the XRD patterns were not observed. As indicated by the Fe contents loaded on biochar (Table S1) and XRD patterns of the pure ferrihydrite^48,52^, iron-containing compounds on the surface or in the pores of Fh@BC were weakly crystalline iron (hydr)oxides^42^.

### As(III) and Cd(II) adsorption in a simultaneous or sequential addition system

#### Adsorption capacity in a single or binary solution

As illustrated in Fig. S3, the order of addition of the As(III) and Cd(II) solutions had a significant effect on their adsorption capacities for As(III) or Cd(II). Among the various treatments, Cd(II) adsorption in the “Cd + As” system and As(III) adsorption in the “As + Cd” system was significantly greater than the other groups (*P* < 0.05). Compared to the single solution, the subsequent addition of As(III) or Cd(II) improved the adsorption of Fh@BC for Cd(II) by 0.29 mg g^−1^ or for As(III) by 0.24 mg g^−1^. However, the pre-equilibrated As(III) or Cd(II) exhibited a significant inhibition effect on the adsorption ability of Fh@BC for subsequently added Cd(II) (− 0.71 mg g^−1^) or As(III) (− 0.37 mg g^−1^) (*P* < 0.05), indicating that As(III) and Cd(II) can compete for the same adsorption sites on the iron (hydr)oxides of modified biochar, which can adsorb the As(III) and Cd(II) simultaneously due to the complexation^9^. Compared to “Cd”, “As” and “Cd and As”, we found that As(III) inhibited the adsorption of Cd(II) in the binary solution (simultaneous addition) on account of the higher complexation constant between As(III) and iron (hydr)oxides than that of Cd(II)^42,53^.

#### Adsorption characteristics in a single or binary solution

**Figure 1:**
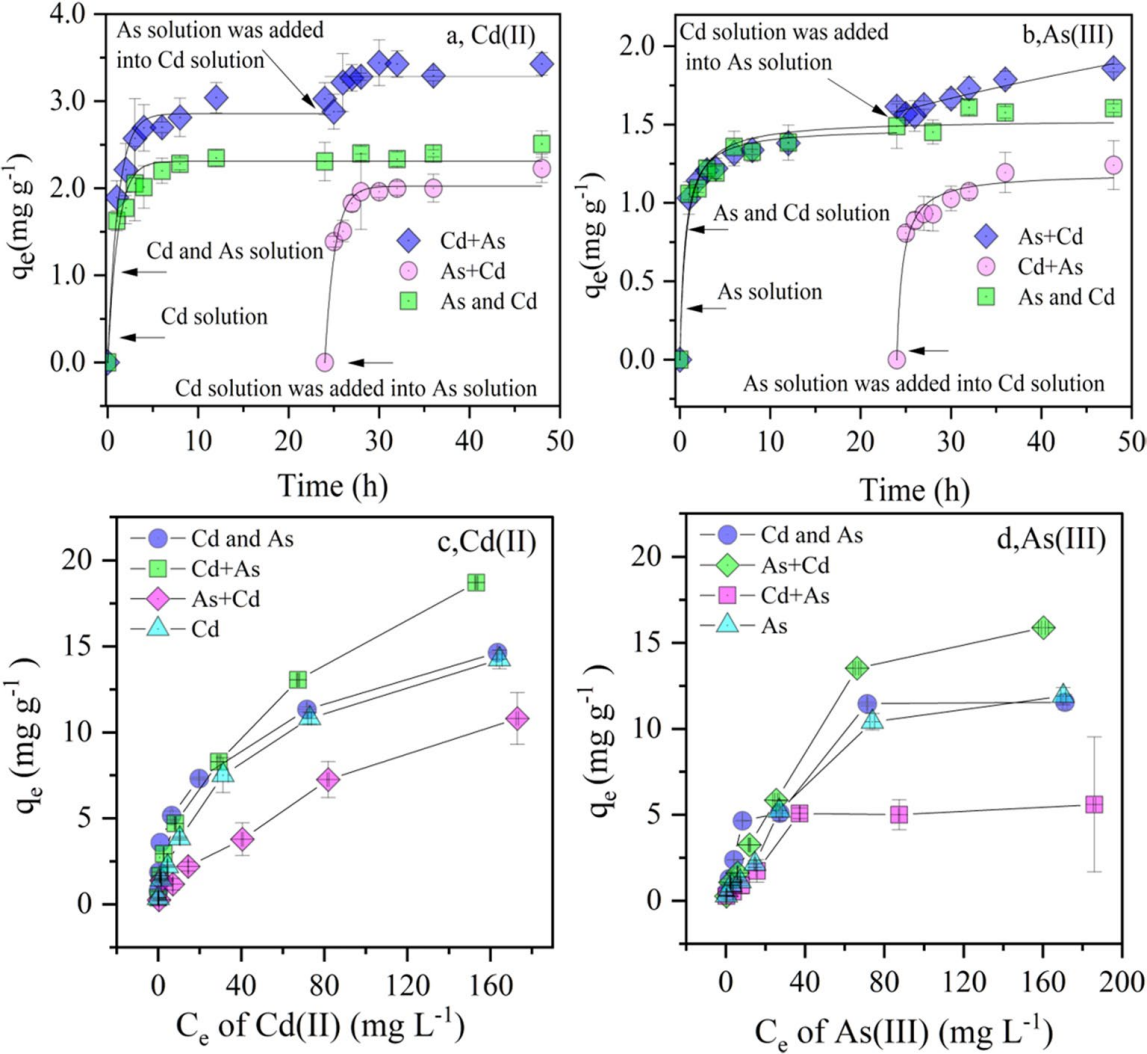
Adsorption kinetics (**a,b**) and isotherm (**c,d**) of As (III) and Cd(II) on Fh@BC in the single or binary solution with pH 7.0 ± 0.1 at 25 ℃.

The adsorption of As(III) and Cd(II) by Fh@BC attained equilibrium within 5 h and 10 h, respectively, as illustrated in Fig. 1a,b. When As(III) or Cd(II) solution was added to the pre-equilibrated Cd(II) or As(III) solution, the adsorption capacity of Fh@BC for Cd(II) or As(III) was improved, indicating that the sequential  addition had a dramatic effect on the adsorption capacity and the adsorption rate of As(III) and Cd(II)^9^. The PSO with a higher R^2^ value much more precisely described the adsorption process of Cd(II) and As(III) in all treatments than the PFO. These results indicated that the removal of Cd(II) and As(III) by Fh@BC was mainly due to the chemical reaction^10^. Further, it appears that the adsorption of Cd(II) and As(III) by Fh@BC was a multiple-step process, which may include external surface diffusion, intraparticle diffusion, and valence forces^5^. Similar results were also found in other studies^5,10^. As can be seen from Table 1, K2 (0.68) of Cd(II) in the “As + Cd” system and *K*_2_ (1.29) of As(III) in the “Cd + As” system were much higher than that of Cd (0.46) and As (1.20) in the single adsorbate solution. This suggested that the pre-equilibrium of As (III) or Cd(II) could improve the adsorption rate of the subsequently added Cd(II) or As(III) in the binary solution.

**Table 1:** Kinetics and isotherms parameters for As (III) and Cd(II) adsorption on Fh@BC (25 °C) in the single solution or binary solution (with a simultaneous or sequential addition).

Parameters	Treatment	Cd				As			
		Cd + As	Cd and As	As + Cd	Cd	Cd + As	Cd and As	As + Cd	As
PFO	*q*_e_ (mg g^−1^)	3.147	2.309	2.022	2.865	1.080	1.442	1.578	1.356
	k_1_ (h^−1^)	0.642	0.909	0.903	0.891	1.055	0.882	0.615	1.165
	*R*^2^	0.918	0.954	0.963	0.973	0.910	0.892	0.833	0.923
PSO	*q*_e_ (mg g^−1^)	3.322	2.426	2.197	3.112	1.190	1.532	1.684	1.481
	k_2_(g (mg h)^−1^)	0.325	0.698	0.679	0.463	1.294	0.954	0.566	1.200
	*R*^2^	0.967	0.989	0.985	0.994	0.965	0.960	0.923	0.968
Langmuir	*K*_L_ (L mg^−1^)	0.021	0.068	0.007	0.026	0.034	0.041	0.017	0.016
	*Q*_m_ (mg g^−1^)	23.712	14.751	19.958	18.181	6.838	13.550	22.809	18.381
	R^2^	0.966	0.890	0.973	0.990	0.913	0.924	0.978	0.970
Freundlich	*K*_F_(mg^(1−n)^ L^n^ g^−1^)	1.763	2.941	0.406	1.420	0.721	1.609	1.033	0.751
	n	2.127	3.178	1.567	2.181	2.412	2.483	1.807	1.804
	R^2^	0.999	0.994	0.982	0.988	0.808	0.896	0.934	0.919

The results of the adsorption isotherm experiments in Fig. 1c,d indicated that the sequential addition of Cd(II) (5 mg L^−1^) or As(III) (5 mg L^−1^) improved the adsorption capacity of Fh@BC for As(III) or Cd(II). Combined with the results in Table 1, it appeared that the Freundlich model provided a better description of Cd(II) adsorption by Fh@BC in the single and binary solution, indicating that the adsorption by Fh@BC included monolayer adsorption and multi-layer adsorption on the biochar surface and iron (hydr)oxides. In contrast to Cd(II), the Langmuir model was a good fit for the adsorption process of As(III) in the single and binary solution, indicating a one-layer distribution on the surface of the Fh@BC^5^.

According to the Langmuir model, Fh@BC displayed a great adsorption capacity for As(III) and Cd(II) in Table 1. The Langmuir maximum adsorption capacity of As(III) and Cd(II) on Fh@BC in the single solution was 18.38 and 18.18 mg g^−1^, respectively. For estimating the adsorption performance, the Langmuir maximum adsorption capacities of Fh@BC for Cd(II) and As(III) were compared with other modified biochars reported in previous studies, as shown in Table S2. Fh@BC, with a simple process of modification, exhibited much more excellent adsorption capacity for simultaneous removal of As(III) and Cd(II) than others^9,54^. This excellent performance of Fh@BC for As(III) and Cd(II) might be explained by the adsorption by PBC and the complexation of iron (hydr)oxides^14,42,55^.

### Mutual effect between As(III) and Cd(II) in a binary solution

**Figure 2:**
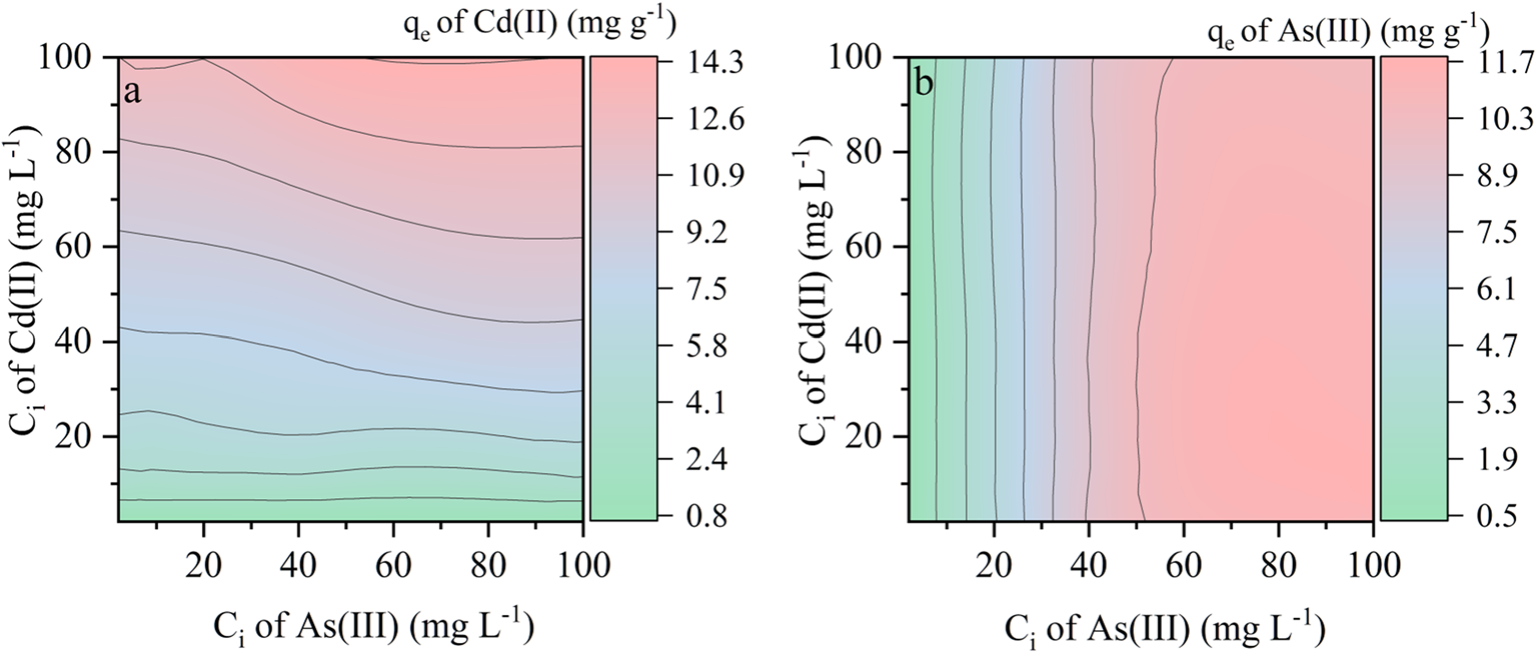
Mutual effect of Cd(II) (C_i_ of 1–100 mg L^−1^) and As(III) (C_i_ of 1–100 mg L^−1^) on adsorption capacities of Fh@BC for Cd(II) (a) and As(III) (b) in the binary system with simultaneous addition.

The mutual effects between As(III) and Cd(II) in binary solution with simultaneous addition were considered in Fig. 2. The results indicated that the adsorption capacity of Fh@BC for Cd(II) was improved by the high concentration of As(III) in binary solution with simultaneous addition in Fig. 2a. However, the simultaneous addition of Cd(II) had little effect on the adsorption capacity of Fh@BC for As(III) in Fig. 2b. As discussed above, it can be concluded that the coexisting As(III) improves the adsorption capacity of Fh@BC for Cd(II) in the binary system.

**Figure 3:**
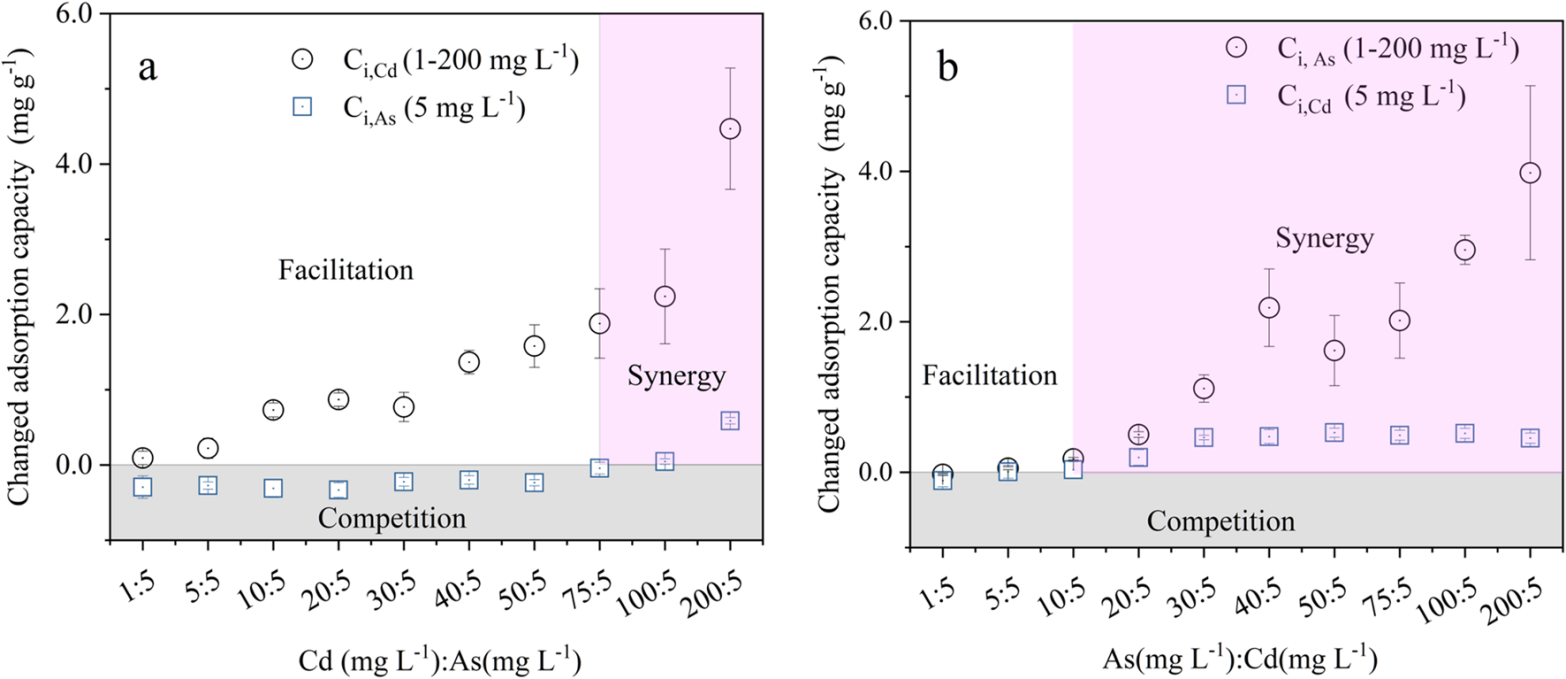
Mutual effect of Cd(II) (C_i_ of 1–200 mg L^−1^) and As(III) (C_i_ of 1–200 mg L^−1^) on adsorption capacities in “Cd + As” system (**a**) and “As + Cd” system (**b**) with sequential addition.

Compared with the “Cd” system, the sequential addition of  5 mg L^−1^ As(III) solution promoted the adsorption of Cd(II) by Fh@BC in the “Cd + As” system by 0.09–4.47 mg g^−1^, as shown in Fig. 3a. In turn, compared to the “As” system, the pre-equilibrated Cd(II) changed the adsorption capacity of Fh@BC for the sequentially added As(III) by -0.30–0.59 mg g^−1^. A synergy effect between As(III) and Cd(II) was observed when the concentration ratio of Cd(II) to As(III) ranged from 75:5 to 200:5. As shown in Fig. 3b, compared with the “As” system, the addition of 5 mg L^−1^ Cd(II) solution also promoted the adsorption of Fh@BC for As(III) in the “As + Cd” system by 0.05–3.98 mg g^−1^. Similar to the “Cd + As” system, a synergy effect between As(III) and Cd(II) in the “As + Cd” system was observed when the concentration ratio of As(III) to Cd(II) ranged from 5:5 to 200:5. Compared to the “Cd” system, the pre-equilibrated As(III) also changed the adsorption capacity of the sequentially added Cd(II) by − 0.11–0.45 mg g^−1^. As(III) and Cd(II) mutually facilitated the adsorption of Fh@BC for Cd(II) or As(III) in binary solution (the "Cd + As" or "As + Cd" system), depending on the ratio of As(III) to Cd(II).

These results indicate that there were competition and promotion between As(III) and Cd(II) on their adsorption capacities on Fh@BC in the binary solution. The interactions depended on the addition sequence of As(III) and Cd(II), which determined the competition between As(III) and Cd(II) on adsorption sites of modified biochar. Wu et al.^9^ demonstrated that the presence of As(III) facilitated Cd(II) adsorption, while the presence of Cd(II) suppressed As(III) adsorption on the modified biochar (calcium-based magnetic biochar). The speculated reason for the facilitation of As(III) on Cd(II) was on account of the electrostatic interaction and the formation of type B ternary surface complexes (=Fe–O–As–O–Cd), and the inhibition of Cd(II) on As(III) was due to the same adsorption sites (i.e., iron (hydr)oxides) on the modified biochar. Other studies and our study have also found competition and facilitation between Cd and As in the binary solution (i.e., the facilitation of As on Cd)^5,10^, and the potential reasons for the facilitation of As(III) on Cd(II) are the electrostatic interaction and the formation of ternary surface complexes (type B)^4,9,56^. Besides, we found an interesting and fresh phenomenon is that Cd(II) could facilitate the adsorption of Fh@BC for As(III) in the binary solution. The speculated reason for the facilitation of Cd(II) and As(III) might include the electrostatic interaction and the formation of type A ternary surface complexes based on the results of Wu et al.^9^ and Liu et al.^4^. When the pH of the solution was 7, the dominated species of As(III) was H_3_AsO_3_ during the batch experiment as shown in Fig. S4. This result demonstrated that electrostatic attraction was not responsible for the As(III) adsorption by Fh@BC in the binary solution; instead, surface complexation between H_3_AsO_3_ and iron (hydr)oxides on Fh@BC likely occurred^4^ and then determined the adsorption capacity of Fh@BC for As(III). Based on this, it further inferred that the facilitation of Cd(II) on As(III) might be a result of the formation of type A ternary surface complexes^56^, and the plan of quantitative evidence should be considered to clarify the contribution of type A in the next work.

### Adsorption mechanism analysis

As mentioned above, Fh@BC could simultaneously adsorb Cd(II) and As(III) in the binary solution, and the potential mechanisms of Fh@BC adsorption for As(III) and Cd(II) are discussed as follows. First, the changes in the pH of the beginning solution (pH_b_) and equilibrium solution (pH_e_) might verify the existence of Fh@BC protonation and deprotonation process during the adsorption process (Fig. S4a and b). The main species of Cd(II) and As(III) in  aqueous solution at pH = 7.0 were Cd^2+^ and H_3_AsO_3_, respectively (Fig. S4c and d). The adsorption of Fh@BC for Cd^2+^ in aqueous solution at pH = 7.0 might be due to electrostatic attraction between Cd^2+^ and negatively charged biochar^26,57^, while the adsorption of Fh@BC for H_3_AsO_3_ at pH = 7.0 could be attributed to the complexation of iron (hydr)oxides^36^. As shown in Fig. S5, ion exchange or electrostatic attraction for Cd(II) adsorption could be identified by the influence of the coexisting cations (Ca^2+^ > Mg^2+^ > K^+^ > Na^+^) ^36^. This indicates that the electrostatic interaction influenced the adsorption performance of Fh@BC for Cd(II), depending on the ionic radius and charge^58^. The co-existence of anions (except H_2_PO_4_^−^) presented a slight effect on the adsorption of  Fh@BC for As(III). This can be attributed to the formation of inner-sphere complexation between As(III) and Fh@BC^52,59,60^. With an increasing H_2_PO_4_^-^ concentration, the adsorption of Fh@BC for As(III) significantly decreased due to the strong competition between H_2_PO_4_^-^ and As(III)^58,61^. Further, Fh@BC exhibited an oxidation capacity for As(III), because ferrihydrite is a natural Fenton reagent that can oxidize As(III) to As(V)^43,44^. The oxidation capacity in this study was about 8.38 mg g^−1^ (Fig. S6).

**Figure 4:**
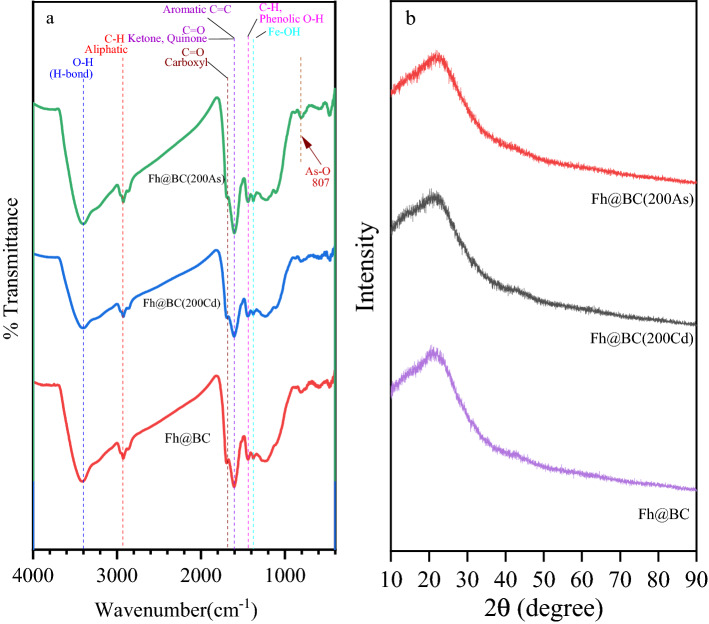
FTIR (**a**) and XRD (**b**) spectrum of Fh@BC before and after the adsorption of As(III) and Cd(II). Fh@BC(200Cd) and Fh@BC(200As) represented the adsorption of Fh@BC for Cd(II) of 200 mg L^−1^ and As(III) of 200 mg L^−1^, respectively.

The FTIR spectrum was presented in Fig. 4a. The weakened characteristic peak of Fh@BC(200Cd) at ~ 3400 cm^−1^ was attributed to Cd(II) complexation with Fe-OH^33^ or R-OH^32,49,50^. The reduction and shift in the characteristic peaks near 1700 and 1600 cm^−1^ indicated that aromatic C=C or C=O of the carboxyl unit were consumed during Cd(II) adsorption^26^, and Cd-π interaction may have occurred^33^. Moreover, the characteristic peak of Fh@BC(200As) at ~ 3400 cm^−1^ was larger as a result of -OH derived from H_3_AsO_3_, despite the existence of complexation between Fe-OH and H_3_AsO_3_^60^. The XRD results of Fh@BC before and after adsorption were shown in Fig. 4b. The absence of well-crystallized minerals indicated that the iron (hydr)oxides on the Fh@BC were still amorphous after adsorption of Cd(II) and As(III). Moreover, the characteristic peaks of As or Cd in the XRD results were not observed. Similar results have been found in other studies that have applied iron-modified biochar for the adsorption of Cd or/and As in the single or binary solution^4,9^.

**Figure 5:**
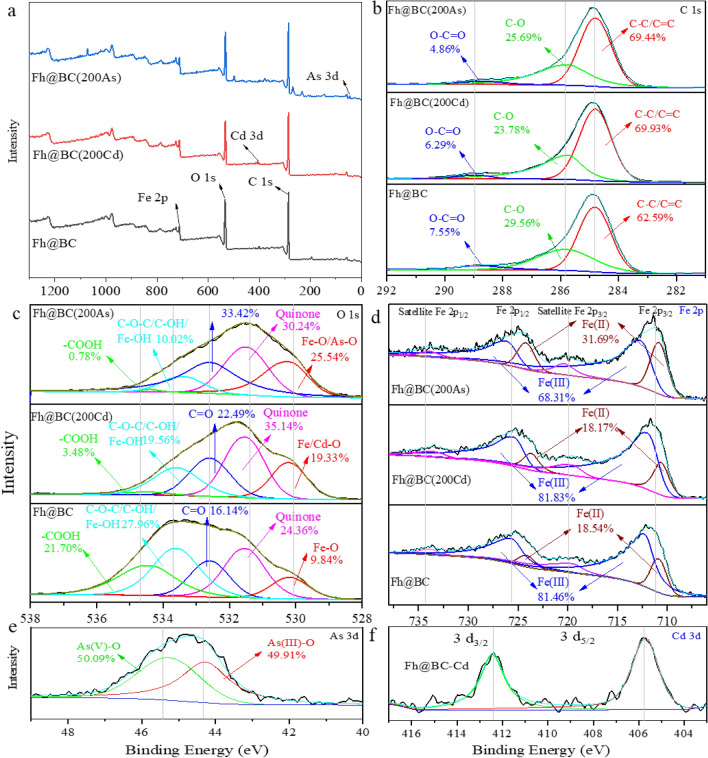
XPS spectrum of Fh@BC before and after the adsorption of As(III) and Cd(II). Fh@BC(200Cd) and Fh@BC(200As) represent the adsorption of Fh@BC for Cd(II) of 200 mg L^−1^ and As(III) of 200 mg L^−1^, respectively. The symbols of (**b**–**f**) represent the XPS spectra of C 1s, O 1s, Fe 2P, Cd 3d, and As 3d, respectively.

C 1s, O 1s, Fe 2P, Cd 3d, and As 3d XPS spectra were used to analyze the evolution of the functional groups on Fh@BC, as illustrated in Fig. 5. The C1s XPS spectrum was divided into three characteristic peaks at the binding energies of 284.8, 286.1 and 288.8 eV, assigned to C–C/C=C, C–O and O–C=O, respectively^33,46^ (Fig. 5b). After As(III) and Cd(II) adsorption, the percentage of C–C/C=C increased from 62.59% to 69.44% and 69.93%, respectively; the ratio of O–C=O decreased from 7.55% to 4.86% and 6.29%, respectively. The shift among the oxygen-containing functional groups indicated that the hydroxyl and carboxyl groups on Fh@BC were involved in the complexation with As(III) and Cd(II) during the adsorption process^26^.

The O1s XPS spectrum was classified into five peaks with binding energies of 530.2, 531.5, 532.6, 533.6 and 534.4 eV, representing metal oxide (M–O), quinone, C=O, C–OH/Fe–OH/C–O–C and –COOH, respectively^31,33,39,62,63^ (Fig. 5c). After adsorption for Cd(II) and As(III), the ratios of C–OH and Fe–OH on Fh@BC-Cd and Fh@BC-As dramatically decreased from 27.96 to 9.56% and 10.02%, respectively; the percentage of –COOH decreased from 21.70 to 3.48% and 0.78%, respectively. These findings indicated that Cd(II) and As(III) could form complexes with C–OH, Fe–OH and –COOH^33^. Moreover, after reacting with As(III) and Cd(II), the relative percentages of Fe–O, Cd–O, and As–O increased from 9.84 to 19.33% and 25.54%. This further confirmed the complexation between contaminants and functional groups.

The Fe 2p was divided into 2p3/2 orbitals of Fe^2+^ and Fe^3+^, 2p3/2 Fe satellite, 2p1/2 orbitals of Fe^2+^ and Fe^3+^, and 2p1/2 Fe satellite, as mentioned by Xu et al.^64^ (Fig. 5d). After the adsorption for Cd(II) and As(III), the percentage of Fe(III) on Fh@BC(200Cd) and Fh@BC(200As) changed from 81.46 to 81.83% and 68.31%, respectively. The shift of Fe 2p on Fh@BC(200As) indicated that Fe(III) on/in Fh@BC was reduced during the adsorption process, this process coupled with oxidation of As(III).

The Cd 3d3/2 and Cd 3d5/2 spectra indicated the existence of Cd–O^35^ (Fig. 5f). As 3d was deconvoluted into two peaks (i.e., As 3d3/2 and As 3d5/2), the proportions of As(III) and As(V) on Fh@BC(200As) were 49.91% and 50.09%, respectively (Fig. 5e). This was supported by the oxidation capacity test of Fh@BC for As(III), which was up to 80.46.% (Fig. S6). Previous studies have demonstrated that iron (hydr)oxides have a strong oxidation capacity for As(III)^33,65^. In addition, As(III) and As(V) generally form bidentate complexes and monodentate complexes with ferrihydrite^42,48^.

**Figure 6:**
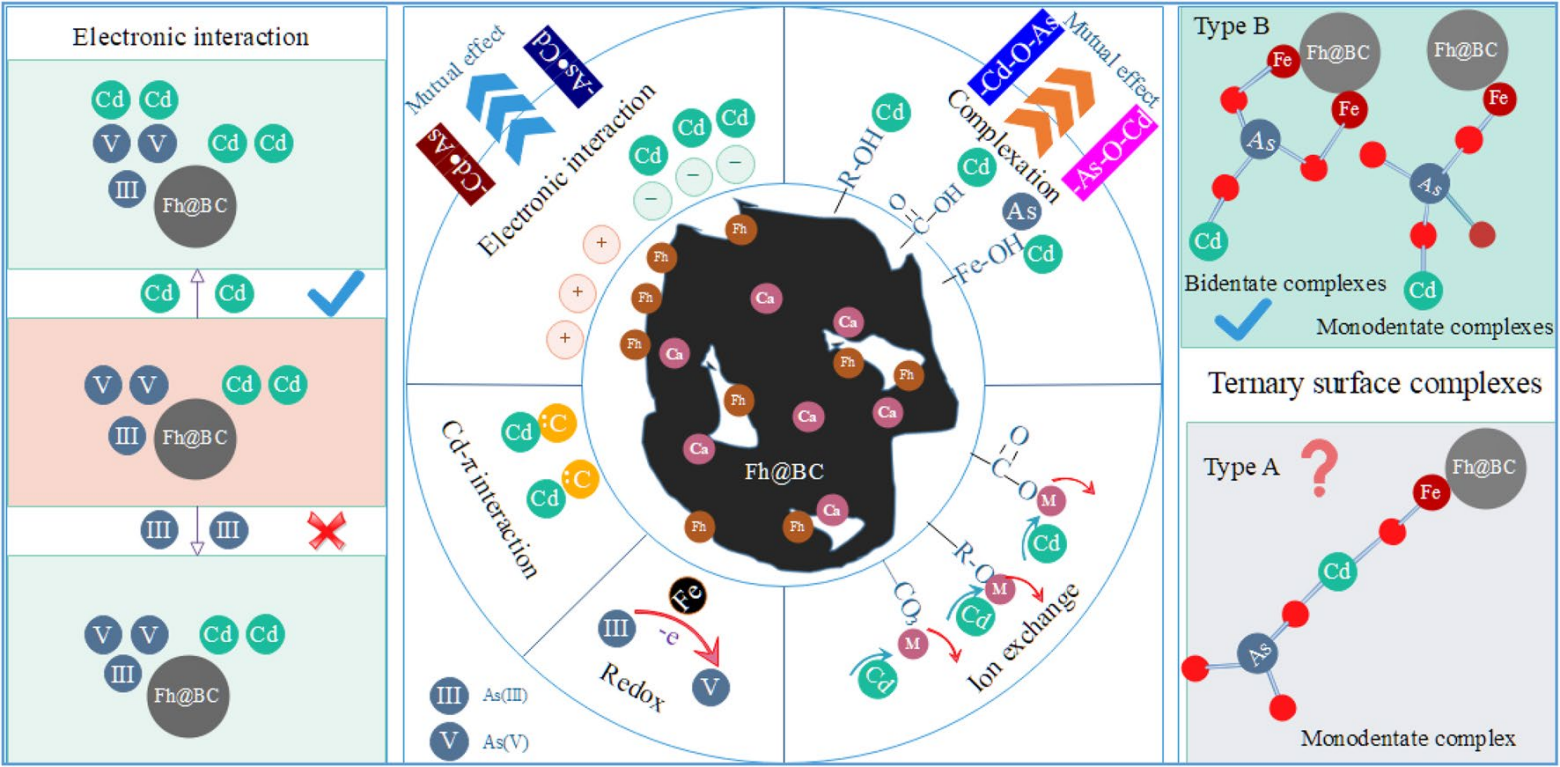
Schematic diagram of adsorption mechanism of Fh@BC for Cd(II) and As(III).

Based on the above, it concluded that Fh@BC could adsorb Cd(II) and As(III) simultaneously in aqueous solution, according to the following potential mechanisms (Fig. 6): (1) cation exchange; (2) complexation of Cd(II) and As(III) with oxygen functional groups, including –COOH, C–OH and Fe-OH; (3) coordination between π-electrons and the C=C of the aromatic structure; and (4) oxidation for As (III). In this paper, the results further confirmed that As(III) could promote the adsorption of Fh@BC for Cd(II), and found a fresh result that the presence of As(III) or Cd(II) could promote the adsorption of Fh@BC to each other (synergy), depending on the concentration ratio and the addition sequence of As(III) and Cd(II). Synergy mechanism between Cd(II) and As (III) in aqueous solution might include the following aspects: (1) electrostatic interaction; (2) the formation of ternary surface complexes (type A or type B).

## Conclusion

In this study, a simple synthetic ferrihydrite-modified biochar (Fh@BC) was applied for the simultaneous removal of As(III) and Cd(II) in aqueous solution. The Langmuir maximum adsorption capacity of Fh@BC for As(III) and Cd(II) in the single adsorbate solution was 18.38 and 18.18 mg g^−1^, respectively. It demonstrated that Fh@BC had the potential for simultaneous removal of As(III) and Cd(II) in aqueous solution. The adsorption mechanisms of Fh@BC for Cd(II) or As(III) mainly included ion exchange and complexation. Moreover, the mutual effect experiment indicated that Cd(II) and As(III) adsorption on Fh@BC in the binary solution exhibited competition, facilitation and synergy, depending on the sequence and concentration ratio of Cd(II) and As(III). The mechanisms of facilitation and synergy of Cd(II) and As(III) in the binary solution might be controlled by electrostatic interactions and type A or type B ternary surface complexes.

## Supplementary Information

Supplementary Information.
