# Supplementary material for: Simultaneous adsorption of As(III) and Cd(II) by ferrihydrite-modified biochar in aqueous solution and their mutual effects
Source: Sci Rep. 2022 Apr 8;12:5918. doi: 10.1038/s41598-022-09648-1 (PMC8993855; doi:10.1038/s41598-022-09648-1)
Supplement: Supplementary file 1 — Supplementary Information. [file 41598_2022_9648_MOESM1_ESM.docx]

**Supplementary information for**

Simultaneous adsorption of As(III) and Cd(II) by ferrihydrite-modified biochar in aqueous solution and their mutual effects

Xiaosong Tian ^a,b,⁎^, Qing Xie ^c^, Guanqun Chai ^d^, Guanghui Li ^b,⁎^

^a.^ College of Resources, Environment and Safety, Chongqing Vocational Institute of Engineering, Chongqing 402260, China

^b.^ Chongqing Engineering Research Center for Soil Contamination Control and Remediation, Chongqing 400067, China

^c^ Interdisciplinary Research Center for Agriculture Green Development in Yangtze River Basin, College of Resources and Environment, Southwest University, Chongqing 400715, China

^d^ Institute of Soil and Fertilizer, Guizhou Academy of Agricultural Sciences, Guiyang 550006, China

^⁎^ Corresponding Authors:

Xiaosong Tian

Address: College of Resources, Environment and Safety, Chongqing Vocational Institute of Engineering, Chongqing 402260, China

E-mail: [terrytian1985@hotmail.com](mailto:terrytian1985@hotmail.com)

Tel: +86-19942319987

Guanghui Li

Address: Chongqing Engineering Research Center for Soil Contamination Control and Remediation, Chongqing 400067, China

Email: [liguanghui@cmhk.com](mailto:liguanghui@cmhk.com)

Tel: +86-18008377708

**Main contents**: Supporting methods (4), figures (6), tables (1).

**Supporting methods**

**SI. 1 Adsorption kinetics:** The adsorption kinetics of Cd(II) and As (III) were described with Eq. (S1) and (S2) ^1,2^:

Pseudo-first-order model:

$$ln\left( q_{e}-q_{t} \right)=\ln q_{e}-k_{1}t (S1)$$

Pseudo-second-order model:

$$\text{t/}\text{q}_{\text{t}}\text{=1/(}\text{k}_{\text{2}}\text{q}_{\text{e}}^{\text{2}}\text{)+ t/}\text{q}_{\text{e}}\text{ }\text{ }\text{ (S2)}$$

where $q_{e}$ and $q_{t}$ is the adsorbed amount of As(III) and Cd(II) on the Fh@BC (mg·g^-1^) at the predicted equilibrium time and at designed time points, respectively; $k_{1}$ (h^-1^) and$k_{2}$ (g·mg^-1^·h^-1^) are the rate constants of the pseudo-first-order and pseudo-second-order models, respectively.

**SI. 2 Adsorption isotherm:** The Langmuir and Freundlich models were adopted to fit the datasets of the adsorption isotherm experiments of Cd(II) and As (III) with Eq. (S3) and (S4) ^2,3^:

Langmuir model:

$$q_{e}= q_{m}K_{L}C_{e}/(1+K_{L}C_{e}) (S3)$$

Freundlich model:

$$\text{q}_{\text{e}}\text{=}\text{K}_{\text{f}}\text{C}_{\text{e}}^{\text{1/n}}\text{ }\text{ }\text{ }\text{ }\text{ }\text{ }\text{ }\text{ }\text{ (}\text{S4}\text{) }$$

Where $q_{e}$ is the equilibrium amount (mg·g^-1^) of Cd(II) and As (III) adsorbed on the surface of Fh@BC; $q_{m}$ is the maximum adsorption amount (mg·g^-1^) corresponding to monolayer adsorption on Fh@BC; $C_{e}$ is the concentration (mg·L^-1^)of Cd(II) and As (III) at equilibrium solution; $K_{L}$ (L∙mg^-1^) and $K_{f}$ (mg^(1-n)^∙L^n^∙g^-1^) are the constants of Langmuir and Freundlich, respectively; 1/n is the constant of the Freundlich model that represents adsorption strength.

**SI. 3 Influencing factors**: The effects of the initial pH on the adsorption efficiency were analyzed in 10 mg·L^-1^ Cd(II) or As (III) solution. The initial pH of the solution was 3-10 for As (III) and 3-8 for Cd(II), respectively, adjusted by 0.1 M HCl or 0.1 M NaOH solution. Distributions of As(III) or Cd(II) species under different pH values in a single adsorbate solution were simulated by Visual MINTEQ 3.1. The effects of coexisting anions (including NO_3_^−^, Cl^−^, SO_4_^2−^, and H_2_PO_4_^−^) and anions (including Na^+^, K^+^, Ca^2+^, and Mg^2+^) on the adsorption of As (III) and Cd(II) were conducted in a single adsorbate solution, respectively. The concentrations of coexisting anions were 0.01 M, 0.05 M, and 0.1 M.

**SI. 4 Oxidation capacity**: The redox experiments were performed with 50±0.1mg Fh@BC in a 20 mL As(III) solution (5-100 mg·L^-1^). The suspension was oscillated in a thermostatic shaker with a rotate speed of 180 rpm at 25℃ for 24 h, and then was filtered through a 0.45 μm filter membrane. The Fh@BC and the filter membrane were desorbed together using 1 M HCl of 20 mL. The solution was measured by HPLC-AFS, and the fresh filter membrane was selected as the control. The As(III) redox in solution and on Fh@BC was determined by the following Eq. (S5)and (S6):

$${RR}_{s}\left( \text{\%} \right)\text{=}\text{C}_{\text{V,s}}/\text{C}_{\text{III+V,s}}\text{ }\text{×100\% }\text{（}\text{S}\text{5}\text{）}$$

$$q_{r,s}\text{=}\text{C}_{\text{V,s}}\times V/M\text{×100\% }\text{ }\text{ }\text{ }\text{ }\text{ }\text{ }\text{（}\text{S}\text{6}\text{）}$$

Where ${RR}_{s}\left( \text{\%} \right)$ is the redox rate of Fh@BC for As(III) to As(V), $\text{C}_{\text{V,s}}$ is the concentration (mg·L^-1^) of the As(V), $\text{C}_{\text{III+V,s}}$ is the concentration (mg·L^-1^) of the sum of As(III) and As(V), $q_{r,s}$ is the adsorbed amounts (mg·g^-1^) of As (V) on Fh@BC, *V* is the volume (mL) of the solution, and *M* is the weight (mg) of the Fh@BC.

**Total figures:6; Total tables：1**

Figure S1 Scanning electron micro-images of Fh@BC under various magnifications of a) 100×, b) 500×, c) 2000×, and d) 50000×.

Figure S2 FTIR (a) and XRD (b) spectrum of PBC and Fh@BC.

Figure S3 Difference in adsorption of Cd(II) and As(III) onto Fh@BC in a single solution and binary solution (with a simultaneous or sequential addition), with three repetitions. The symbols of “A-C” represent the significant difference at 0.05 level based on Turkey-test.

Figure S4 Initial pH (pH_i_) and equilibrium pH (pH_e_) during the adsorption process of Fh@BC for Cd(II) (a) and As(III) (b); Species of 10 mg·L^-1^ Cd(II) (c) and 10 mg·L^-1^ As(III) (d) in 0.01M NaNO_3_ electrolyte solution with various pH.

Figure S5 Effects of coexisting cations (Na^+^, K^+^, Mg^2+^, and Ca^2+^) on Cd(II) adsorption (a), and coexisting anions (Cl^−^, SO_4_^2−^, and H_2_PO_4_^−^) on As(III) adsorption (b) in a single solution. The concentrations of coexisting cations and anions were 0.01 M, 0.05 M and 0.1 M, with three repetitions.

Figure S6 Oxidation capacity of Fh@BC for As(III) with three repetitions. “a” represents the percentage of As species in initial solution (IS), equilibrium solution (ES), and on/in the Fh@BC (AM) in a single adsorbate solution (10 mg·L^-1^ for As(III)); “b” represents the oxidation capacity of Fh@BC for As(III) in a single adsorbate solution.

Table S1 Various parameters of PBC and Fh@BC.

Figure S1


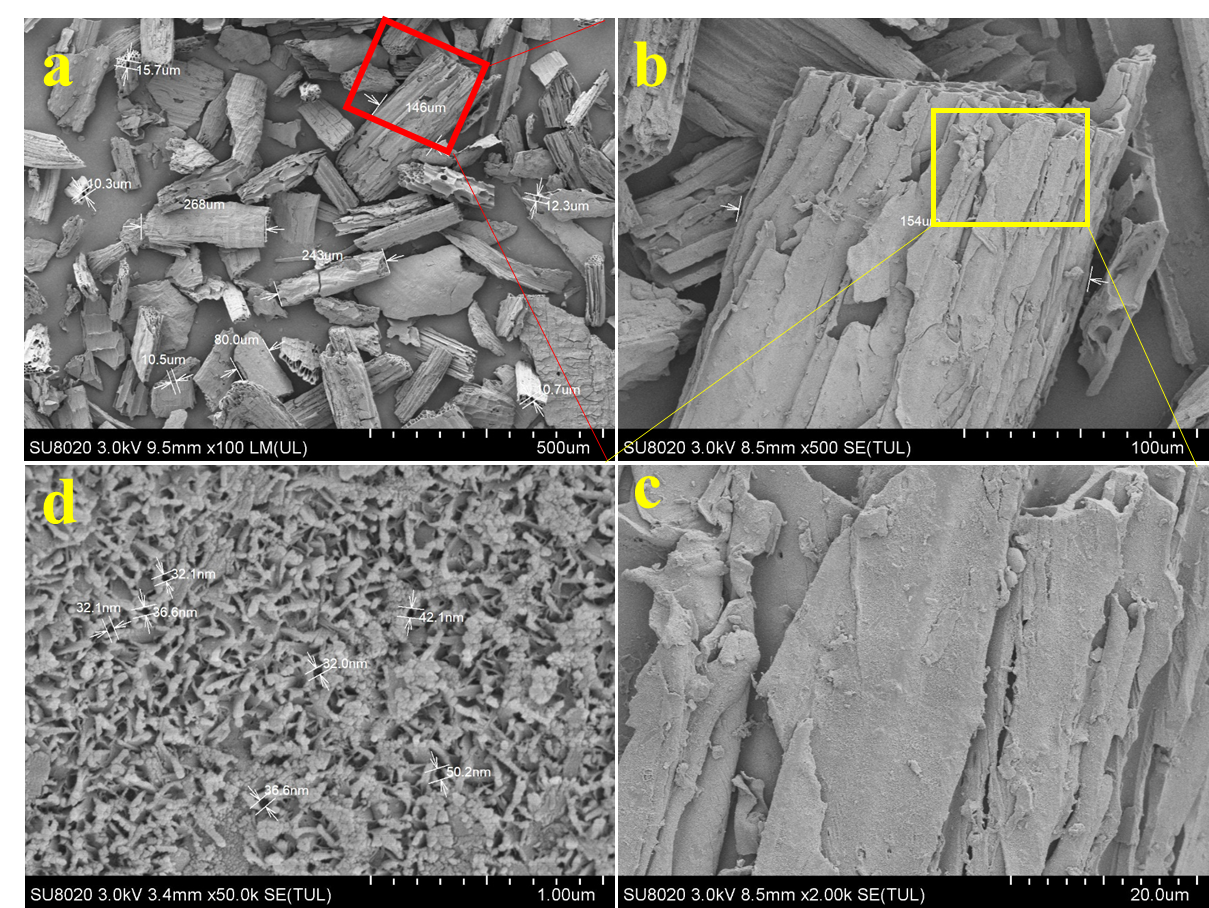


Figure S2

Figure S3


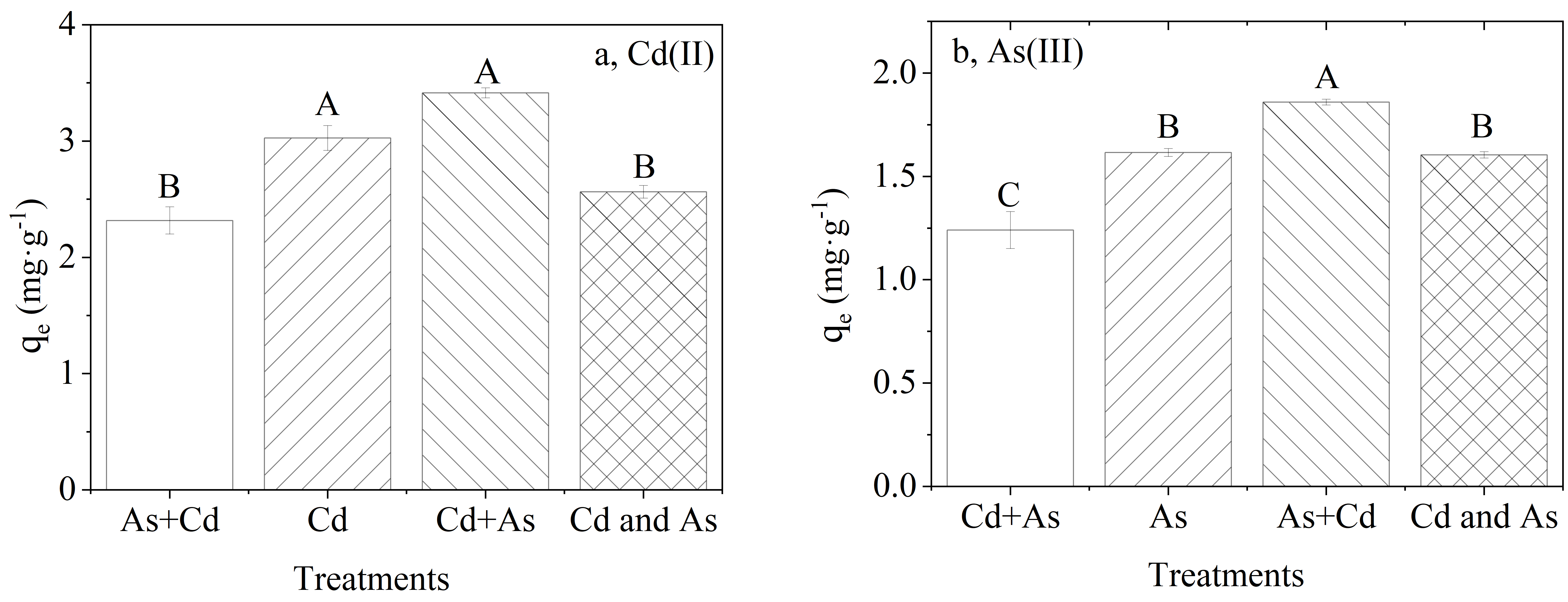


Figure S4


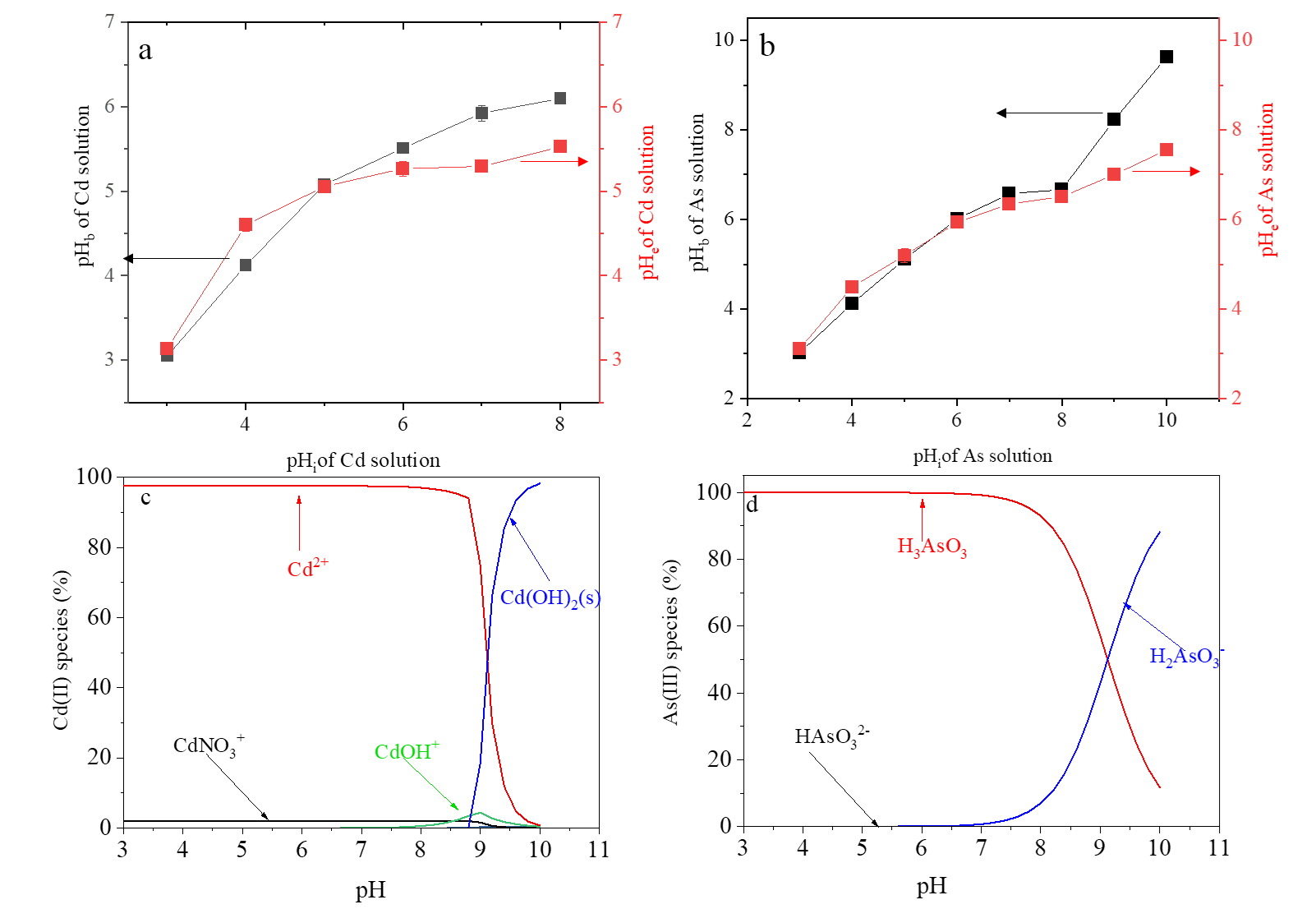


Figure S5


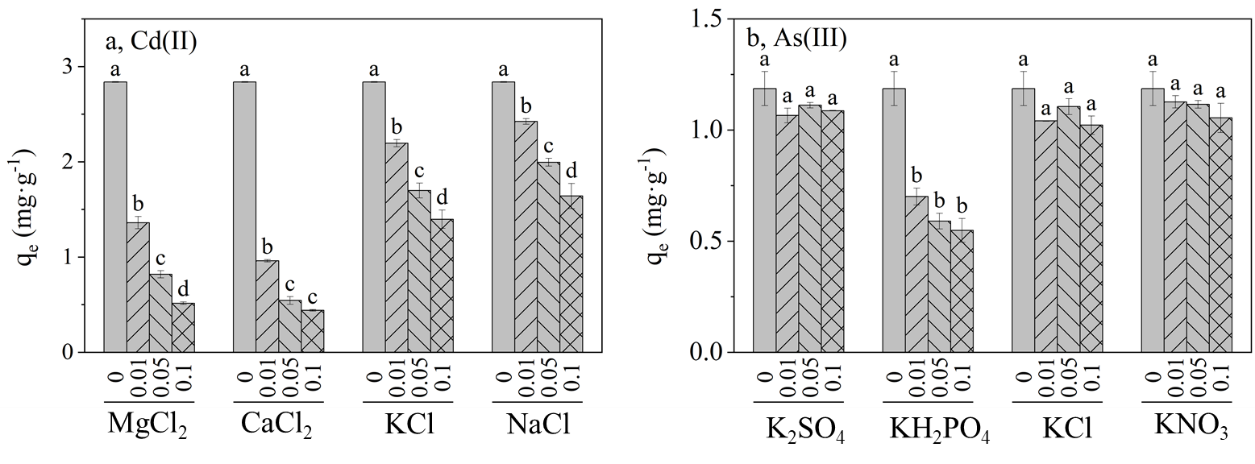


Figure S6


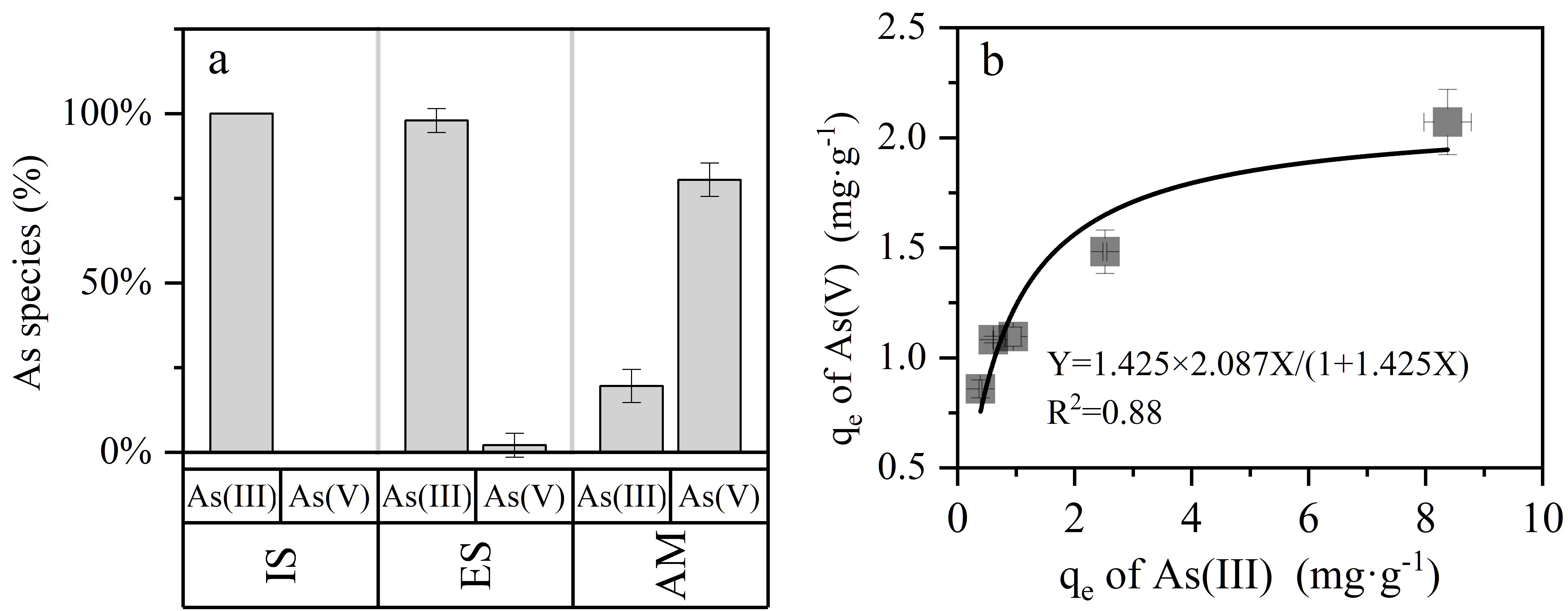


Table S1

| Parameters | PBC (n=3) | | Fh@BC (n=3) | |
| --- | --- | --- | --- | --- |
|  | Mean | SD | Mean | SD |
| pH ^a^ | 9.61 | 0.07 | 6.78 | 0.06 |
| Fe (g/kg) | 34.98 | 0.10 | 72.28 | 1.54 |
| Ca (g/kg) | 193.46 | 60.68 | 46.85 | 1.01 |
| Cd(mg/kg) | ND | ND | 0.23 | 0.09 |
| As(mg/kg) | ND | ND | ND | ND |
| Fe(g/kg) ^b^ | 0.04 | 0.03 | 47.36 | 1.89 |
| BET(m^2^/g) | 3.76 | 0.01 | 4.13 | 0.03 |

“a” represents the pH determined with the solid-liquid ratio of 1g (PBC) to 20mL (ultrapure water).“b” represents the Fe concentration loaded on the PBC determined by the ASS with three repetitions.ND represents the concentration of elements undetected by the XRF with three repetitions.

**References**

1 Zhou, Z. *et al.* Sorption performance and mechanisms of arsenic(V) removal by magnetic gelatin-modified biochar. *Chemical Engineering Journal* **314**, 223-231, doi:10.1016/j.cej.2016.12.113 (2017).

2 Zhu, S. *et al.* Goethite modified biochar as a multifunctional amendment for cationic Cd(II), anionic As(III), roxarsone, and phosphorus in soil and water. *Journal of Cleaner Production* **247**, 119579, doi:10.1016/j.jclepro.2019.119579 (2020).

3 Yang, F. *et al.* Assembling biochar with various layered double hydroxides for enhancement of phosphorus recovery. *Journal of Hazardous Materials* **365**, 665-673, doi:10.1016/j.jhazmat.2018.11.047 (2019).
